# Locating the Origin of Femoral Maltorsion Using 3D Volumetric Technology—The Hockey Stick Theory

**DOI:** 10.3390/jcm9123835

**Published:** 2020-11-26

**Authors:** Joan Ferràs-Tarragó, Vicente Sanchis-Alfonso, Cristina Ramírez-Fuentes, Alejandro Roselló-Añón, Francisco Baixauli-García

**Affiliations:** 1 Department of Orthopaedic Surgery, Hospital Universitarioy Politécnico La Fe, 46026 Valencia, Spain; cotferras@gmail.com (J.F.-T.); baixauli_fragar@gva.es (F.B.-G.); 2 Department of Orthopaedic Surgery, Hospital Arnau de Vilanova, 46015 Valencia, Spain; alexrosello82@gmail.com; 3 Department of Radiology, Hospital Universitario y Politécnico La Fe, 46026 Valencia, Spain; crisramirezfuentes@gmail.com

**Keywords:** femoral anteversion origin, femoral osteotomy, anterior knee pain

## Abstract

Background: The origin of femoral maltorsion is often unknown. However, defining the origin of the rotation of the femoral maltorsion can be useful for establishing the most suitable point to do an external derotational osteotomy. Previous studies have not considered the femoral diaphysis in their investigations of the origin of the deformity. The study of the whole morphology of the femur with 3D volumetric tools, including the femoral diaphysis can contribute to a better understanding of the behavior of femoral maltorsion. Methods: An atypical case of unilateral femoral anteversion was selected. Both femurs were used to obtain 3D bio-models. The mirror image of the asymptomatic side was obtained and overlapped with the symptomatic femur. The Hausdorff–Besicovitch method was used to evaluate the morphologic discrepancies (in mm) between the two femurs in three zones: (1) the femoral neck, (2) the proximal diaphysis, and (3) the distal diaphysis. The differences between the two femurs were analyzed and its correlation was statistically defined using a lineal regression model. Results: The deformity in the distal diaphysis increased from the supracondylar area until the apex of the antecurvatum angle (*R*^2^ = 0.91) and then decreased until the base of the femoral neck (*R*^2^ = (−0.83)), to finally increase significantly in the femoral neck area (*R*^2^ = 0.87). All of the correlations were statistically significant (*p-*value ˂ 0.001). Conclusion: The femoral maltorsion originates in the supracondylar area and its rotational axis is the longitudinal axis of the femoral diaphysis. Even though the deformity affects the femoral diaphysis, its clinical relevance is much higher in the femoral neck since the rotational axis passes through its base. Thus, the osteotomy can be conducted along all of the femoral diaphysis as long as it is done perpendicular to it.

## 1. Introduction

Derotational femoral osteotomies may be indicated in patients with femoral maltorsion with disabling anterior knee pain (AKP) that is unresponsive to conservative treatment [1,2,3]. The first step in performing a corrective osteotomy of a deformity is to establish its location and magnitude. Currently, only the total torsion is measured [4], which does not allow the differentiation of a torsional abnormality located in the proximal or distal femur from one in the diaphysis. Furthermore, there is no evidence to guide decisions regarding the level of osteotomy, and surgery is often performed based to the surgeon’s experience [5]. However, in theory, the osteotomy should be performed at the level of the deformity. Performing an osteotomy anywhere other than the origin of the deformity essentially corrects the malformation by generating a new deformity of the same magnitude and in the opposite direction; that is, it creates a deformity that is double compensated. 

Several authors have used conventional imaging studies in their attempts to define where the torsion occurs along the length of the femur [6,7,8]. Currently, thanks to advances in three-dimensional (3D) software, volumetric and biometric studies can be performed. A previous study demonstrated that the left and right femurs of healthy adults are highly symmetrical [9]. This finding has led to the hypothesis that the contralateral femur could be used as a reference for preoperative templating. In this study, our main objective was to present a new protocol for planning derotational femoral osteotomy. This protocol emphasizes locating the deformity by using the new techniques of volumetric reconstruction and 3D superposition.

## 2. Experimental Methods

**Figure 1:**
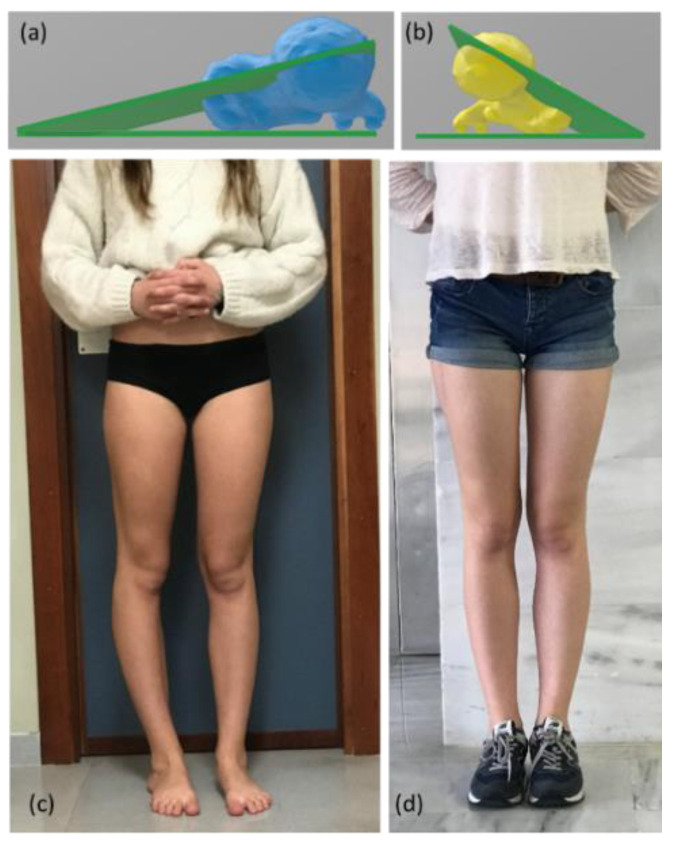
Image of the measurement of the femoral anteversion of the healthy side ((**a**), in blue) and the pathological side ((**b**), yellow) using the technique described by Georgiadis in a 3D program [7]. Both femurs take the femoral neck axis and the posterior intercondyle line as a reference to measure the femoral torsion. (**c**) Clinical pre-operative image. The right leg shows a clear torsion abnormality. (**d**) Post-operative image. Both legs were properly aligned. Clinically, the patient is completely asymptomatic.

The study is based on the case of an 18-year-old patient with pathological right femoral anteversion (39° according to the Murphy method [10], 35° based on the method proposed by Georgiadis, which uses 3D reconstruction [7]) (Figure 1). The patient had disabling right hip pain (VAS 8) and right AKP (VAS 7), with AKP having appeared several months after the onset of the hip pain. The skeletal alignment of the lower left limb was correct (femoral anteversion of 21° according to the Murphy method [10] and 15° using 3D technology [7]) (Figure 1). The right external tibial torsion was 44° and the left was 35°. Both the left hip and knee were completely asymptomatic. Therefore, we considered the left femur as “normal” for this patient and used the left femoral morphology as the reference in planning the osteotomy. A 3D-CT analysis of femoral symmetricity in 15 patients (30 3D bio-models) was performed previously in order to validate the usefulness of the Haussdorf–Besicovitch method in evaluating symmetricity. It has been demonstrated that intrapersonal femoral asymmetry is low enough to use the mirror image of the healthy side as a reference for three-dimensional surgical planning [11].

A computed tomography (CT) scan of both femurs was performed (BrillanceiCT scanner, Philips Medical Systems, Amsterdam, Holand). The patient was placed in the supine position. Then, a single image was acquired during the arterial phase with a scan from the pelvis proximally through the patient’s proximal tibia distally. There was a scheduled start to detect a ROI of 120 HU (Hounsfield units) and an additional delay of 15 s. The images were acquired with 120 Kv and 220 mAs, a 1-mm slice thickness, a 0.5 mm reconstruction interval and a soft tissue window.

**Figure 2:**
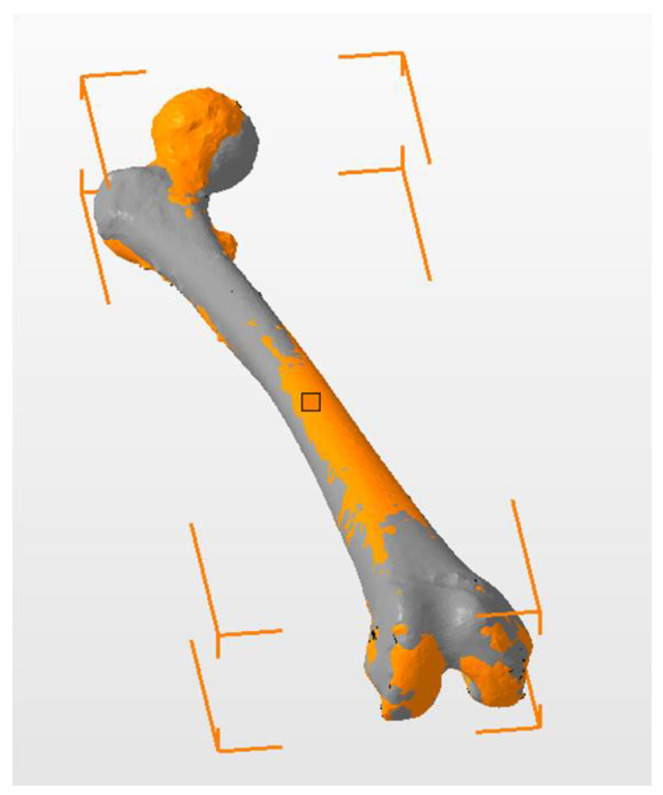
Manual overlap of both femurs. The healthy side is gray, and the pathological side is orange. The color code of this image does not represent a quantitative scale of differences. The aim of this image is to show where the two femurs overlap.

**Figure 3:**
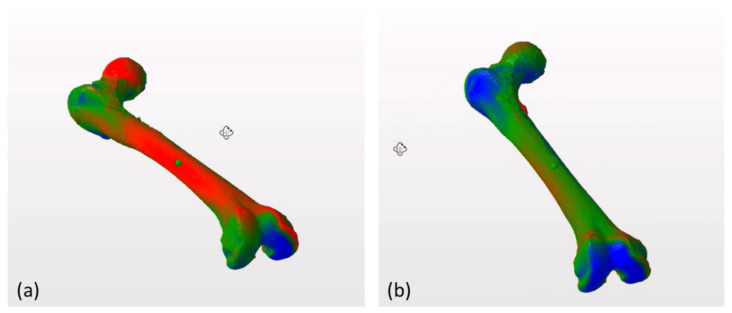
Overlapping of both femurs of one patient using automatic alignment tools (**a**). Manual alignment of the same femurs using the horizontal plane as a common reference point, following the table top method [13] (**b**). The pattern of discrepancies between femurs changes significantly depending on the alignment tool used. In the image on the right (**b**) the similarity between both femurs is greater than in the image on the left (**a**) because they are aligned using common anatomical landmarks for comparison, which allows us to detect areas of anatomical variability between both structures.

The 3D model was obtained after rendering (3D Slicer^®^ version 4.6.2, 3D Slicer Organization—Meshmixer^®^ version 3.5.474, Autodesk, San Rafael, CA, USA). The specular image of the left femur was obtained for comparison with the right femur (3D Builder, Microsoft Corporation^®^ Redmond, WA, USA, version 18.0.1931.0). Then we drew a horizontal plane and located the contact points of the two femurs in the horizontal plane (MeshLab^®^ Visual Computing Laboratory, Pisa, Italy) [12]. This process enabled us to manually select the connecting points of both femurs. We used the horizontal plane because it is one of the planes used to define the femoral anteversion. Then, we manually overlapped the images of the two femurs (Figure 2). In a previous study, we analyzed the great importance of overlapping the images manually [11] (Figure 3).

**Figure 4:**
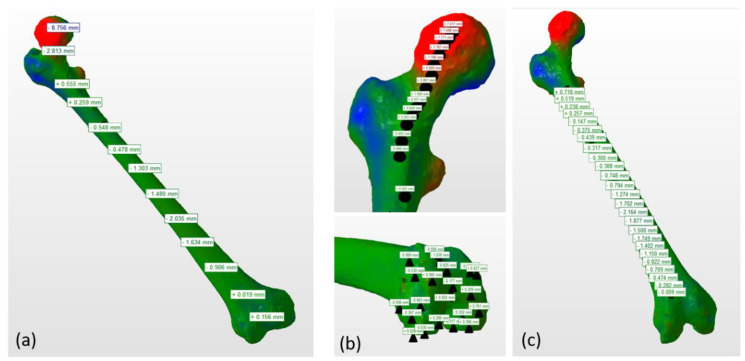
Measurement of the discrepancy between two femurs along the longitudinal axis of the femur. (**a**) General view. (**b**,**c**) Details of the proximal femur, femoral diaphysis, and distal femur. Note that the two distal femurs match almost perfectly, while the diaphyseal and proximal femurs do not match due to the rotational deformity. Color legend: the more green the color is, the less difference between both femurs. The redder the color is, the greater the positive difference between both femurs and the more blue the color is, the greater the negative difference between both femurs. After aligning both femurs manually, reference points were noted at a regular interval distance as shown in the images.

To ensure that both femurs were properly superimposed, we used two reference points distally and one proximally to create a triangular system in the same horizontal plane. Once the femurs were superimposed, the discrepancy in the longitudinal axis of the femur was measured in millimeters from one femur to the other along the entire length, with a constant distance of 20 mm from one measuring point to the next in the diaphysis and 5 mm in the femoral neck (Netfabb^®^ Autodesk, San Rafael, CA, USA) (Figure 4). In this way, the larger the difference in millimeters between the femurs at a measuring point, the less anatomical similarity of the femurs. The congruence between both femurs at the level of the posterior condyles and the proximal support area on the greater trochanter (differences less than 0.05 mm) was verified to be perfect. A linear regression analysis of the progression of the distal deformity to the intertrochanteric line and proximal to the intertrochanteric line was performed independently to assess the incongruence at the femoral diaphyseal level and at the proximal femoral level.

## 3. Results

**Figure 5:**
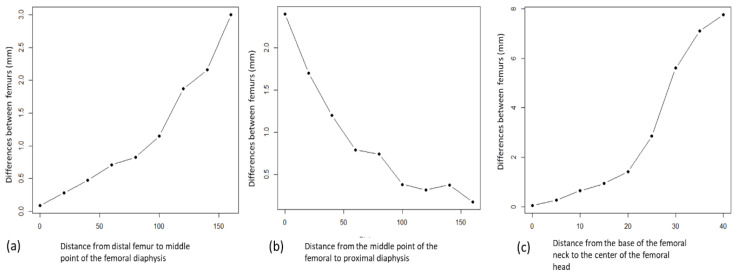
(**a**) Progressive increase of the deformity at the level of the distal femoral diaphysis to its apex, at the middle third level of the femoral diaphyseal, with lineal behavior (*p* < 0.01). The intervals between measurements are regular so the increase in the discrepancies between the measured points is due to the increase in the asymmetry between the femurs. (**b**) Progressive reduction of the deformity between the proximal femoral diaphysis level from its middle point, at the middle third level of the femoral diaphyseal and the proximal femur, with lineal behavior (*p* < 0.01). (**c**) Progressive increase of the deformity at the femoral neck level from the base, with lineal behavior (*p* < 0.01).

A trimodal pattern of femoral deformity was found between the healthy side and the pathological side. At the level of the femoral diaphysis, the deformity increased linearly from the distal metaphysis to the diaphyseal middle zone (Figure 5a). 

It subsequently decreased from the diaphyseal middle zone to the proximal diaphysis, with a statistically similar linear pattern (Figure 5b). 

The progression of the deformity at the level of the distal femoral diaphysis showed a progressively increasing linear component (*R*^2^ = 0.91, F1, 4 = 84.1) that was statistically significant (*p* < 0.01). At the proximal diaphyseal level, there was a statistically significant decreasing linear correlation (*R*^2^ = 0.83, F1, 7 = 40.49, *p* > 0.01). At the neck level, the third deformity pattern demonstrated a statistically significant increasing progressive linear correlation that was greater than in either the proximal or the distal diaphysis (*R*^2^ = 0.87, F1, 7 = 56.51, *p* < 0.01) (Figure 5c).

## 4. Discussion

Here, we present a new protocol using the new techniques of volumetric reconstruction and 3D superposition to locate the deformity in order to plan a derotational femoral osteotomy. Other authors have used volumetric structure superposition programs to assess femoral morphology [9], but the automatic structure superposition algorithms do not allow for the evaluation of the origin of femoral anteversion.

Femoral anteversion is defined by the angle formed by the intersection of two reference lines: one proximal that represents the axis of the femoral neck, and one distal that is tangential to the posterior aspect of the femoral condyles. When this angle is pathological, there is a torsional alteration of the femur; however, it is not possible to specify the level of this torsional alteration. Defining the level of the torsional alteration requires defining three or four reference lines. Kim et al. defined a new axis on CT that extended from the center of the lesser trochanter to the center of the greater trochanter [8]. They referred to this axis as the intertrochanteric line (ITL); however, it is unclear which CT images were selected to place the lesser and greater trochanters on this line. The authors divided the total femoral torsion into a portion below the ITL (the infra-trochanteric torsion) and the portion above the ITL (the supra-trochanteric torsion). Seitlinger et al. drew two axes in the transverse plane, one at the level of the lesser trochanter and one using the flat surface of the distal supracondylar femur [14]. This approach produced three angular measurements: (a) the proximal torsion between the lesser trochanteric line and the femoral neck axis, (b) the shaft torsion between the lesser trochanteric line and the supracondylar line, and (c) the distal femoral torsion between the supracondylar femur and the femoral condyles. In short, torsion was identified in the femoral shaft, above the femoral shaft, and below the femoral shaft. In the control group, the amount of torsion above the shaft was only 2.5° more than the amount of torsion in the shaft. In contrast, the anteversion group had more internal torsion above the femoral shaft (35°) and less external torsion in the femoral shaft (19°).

The greater trochanter may not have a constant shape and location, therefore, the only well-defined points for drawing the additional reference lines are the lesser trochanter and the center of the femoral diaphysis. However, using the center of the lesser trochanter raises the following question of whether the lesser trochanter occupies a constant position on the proximal femur? The possibilities of placing torsion above or below the lesser trochanter would not be appropriate if its position changes. Therefore, other methods for locating the origin of the torsional abnormality are necessary. The new techniques of volumetric reconstruction and 3D superposition could be suitable for locating the origin of the torsional abnormality. In our model, we did not use automatic overlapping tools because the alignment between both femurs would be unpredictable. Instead, our model establishes a common reference plane that is one of the planes used in the anatomical definition of femoral anteversion. The use of this plane is the only way to establish that the differences between the two femurs being compared are due to the deformity and not to the alignment because it is controlled and therefore known.

**Figure 6:**
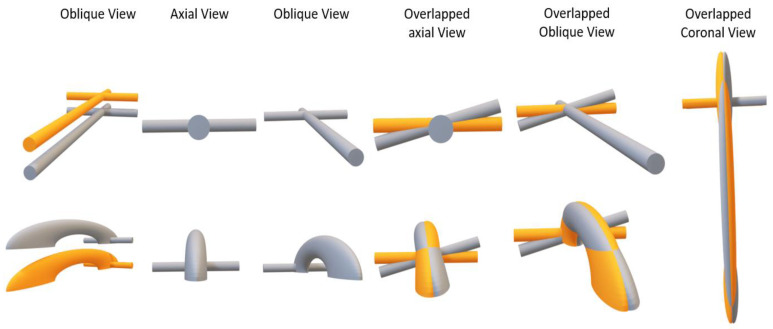
Hockey stick theory. Superior: if the femoral diaphysis was a cylinder, the rotation in the axial plane would not affect the morphology of the femoral diaphysis. The axial rotation would increase the discrepancies between the two femurs in the femoral neck but not in the diaphysis. Inferior: the femur has a certain degree of antecurvatum. Thus, differences in the axial rotation of the diaphysis can be revealed, with the maximum difference in the apex of the antecurvatum angle (Video S1).

Our data show a bimodal deformity in the femoral diaphysis originating at the supracondylar level, and a more evident deformity at the level of the proximal femur at the neck and femoral head; therefore, the femoral anteversion of our particular patient is a trimodal deformity. The diaphyseal deformity occurs in a progressive way from the supracondylar zone to the middle diaphyseal zone. It subsequently recedes to return to congruence just at the beginning of the base of the femoral neck. It then initiates a deformity that progresses in a more pronounced way, reaching its maximum in the femoral head. The study of the 3D morphology of the femoral diaphysis was important to determine the origin of the deformity. Due to the femoral antecurvatum, rotational diaphyseal abnormalities can be detected by our method (Figure 6). Previous studies, such as that of Kim et al. [8], do not include the femoral diaphysis in their analysis of the origin of the deformity, which is a limitation of Kim´s study.

## 5. Clinical Relevance

Certain clinical questions are currently unanswered: How important is the level of the osteotomy? Should the pattern of torsion distribution determine the level of the osteotomy? Knowing where the torsional alteration originates could be clinically relevant since it would identify where to make the osteotomy correction. For example, Seitlinger et al. showed more torsion from above the shaft in their high anteversion group, which would support rotational correction in the proximal femur [14]. This finding coincides with our results.

## 6. Limitations

An important limitation of our method is that it is only valid in cases of primary unilateral torsional abnormalities, which is uncommon, at least in our daily clinical practice. That is, we need a contralateral lower limb with a normal skeletal alignment that can serve as a basis for comparison. This technique would obviously have no meaning in post-fracture femoral torsional alterations because it is clear where the origin of the torsional alteration lies in these cases. 

Finally, due to the low prevalence of unilateral femoral torsion abnormalities, this study was performed only in one patient with unilateral femoral maltorsion. The purpose of this work is to present a new theory that is compatible with our data and a bibliography regarding the behavior of the femoral torsion. However, we need more studies using this methodology with more patients to confirm the hockey stick theory. Due to the low prevalence of this condition, we encourage other authors to contribute by using their patients to analyze this new theory.

## 7. Conclusions

Three-dimensional technology and advanced techniques to assess similarities between volumetric structures provide a good method for planning derotational femoral osteotomy in patients with unilateral torsional femur abnormalities.

## Supplementary Materials

The following are available online at https://www.mdpi.com/2077-0383/9/12/3835/s1, Video S1: Hockey stick theory about the origin of the femoral torsion.
